# Development and evaluation of different electroactive poly(vinylidene fluoride) architectures for endothelial cell culture

**DOI:** 10.3389/fbioe.2022.1044667

**Published:** 2022-10-19

**Authors:** David Durán-Rey, Ricardo Brito-Pereira, Clarisse Ribeiro, Sylvie Ribeiro, Juan A. Sánchez-Margallo, Verónica Crisóstomo, Igor Irastorza, Unai Silván, Senentxu Lanceros-Méndez, Francisco M. Sánchez-Margallo

**Affiliations:** ^1^ Jesús Usón Minimally Invasive Surgery Centre, Cáceres, Spain; ^2^ CMEMS-UMinho, University of Minho, Guimarães, Portugal; ^3^ LABBELS-Associate Laboratory, Braga/Guimarães, Portugal; ^4^ CF-UM-UP, Physics Centre of Minho and Porto Universities, University of Minho—Campus de Gualtar, Braga, Portugal; ^5^ IB-S Institute of Science and Innovation for Bio-Sustainability, University of Minho, Campus de Gualtar, Braga, Portugal; ^6^ LaPMET—Laboratory of Physics for Materials and Emergent Technologies, University of Minho, Braga, Portugal; ^7^ Centro de Investigación Biomédica en Red de Enfermedades Cardiovasculares (CIBERCV), Instituto de Salud Carlos III, Madrid, Spain; ^8^ RICORS-TERAV Network, Instituto de Salud Carlos III, Madrid, Spain; ^9^ Cell Biology and Histology Department, Faculty of Medicine, Leioa, Spain; ^10^ BCMaterials, Basque Center for Materials, Applications and Nanostructures, UPV/EHU Science Park, Leioa, Spain; ^11^ Ikerbasque, Basque Foundation for Science, Bilbao, Spain

**Keywords:** PVDF, films, membranes, electrospinning, tissue engineering, scaffolds

## Abstract

Tissue engineering (TE) aims to develop structures that improve or even replace the biological functions of tissues and organs. Mechanical properties, physical-chemical characteristics, biocompatibility, and biological performance of the materials are essential factors for their applicability in TE. Poly(vinylidene fluoride) (PVDF) is a thermoplastic polymer that exhibits good mechanical properties, high biocompatibility and excellent thermal properties. However, PVDF structuring, and the corresponding processing methods used for its preparation are known to significantly influence these characteristics.

In this study, doctor blade, salt-leaching, and electrospinning processing methods were used to produce PVDF-based structures in the form of films, porous membranes, and fiber scaffolds, respectively. These PVDF scaffolds were subjected to a variety of characterizations and analyses, including physicochemical analysis, contact angle measurement, cytotoxicity assessment and cell proliferation.

All prepared PVDF scaffolds are characterized by a mechanical response typical of ductile materials. PVDF films displayed mostly vibration modes for the a-phase, while the remaining PVDF samples were characterized by a higher content of electroactive β-phase due the low temperature solvent evaporation during processing. No significant variations have been observed between the different PVDF membranes with respect to the melting transition. In addition, all analysed PVDF samples present a hydrophobic behavior. On the other hand, cytotoxicity assays confirm that cell viability is maintained independently of the architecture and processing method. Finally, all the PVDF samples promote human umbilical vein endothelial cells (HUVECs) proliferation, being higher on the PVDF film and electrospun randomly-oriented membranes. These findings demonstrated the importance of PVDF topography on HUVEC behavior, which can be used for the design of vascular implants.

## 1 Introduction

Properties of the extracellular matrix (ECM) such as porosity, stiffness and architecture strongly affect cell behavior. Therefore, there is a need to control these parameters in scaffolds and materials intended for tissue engineering (TE) applications (Khorshidi et al., 2015). A large number of materials have been proposed for the repair of different tissues or organs, such as cardiac (Weinberger et al., 2017), gastrointestinal (Penkala and Kim, 2007), vascular (Durán-Rey et al., 2021), nerve (Motamedi et al., 2017a), and bone tissues (Fernandes et al., 2019). Characteristics, such as their mechanical properties, biocompatibility and physicochemical characteristics, together with the biological performance of the scaffolds, are essential to ensure an optimal cell response.

Active and smart materials are increasingly being implemented for advanced tissue regeneration strategies, being piezoelectric materials of particular interest, as they can generate an electrical potential in response to an applied stress or mechanical deformation (Motamedi et al., 2017b; Li et al., 2019; Mokhtari et al., 2021). Considering that electric stimulation has shown great promise for the development of materials for TE by mimicking the dynamic electroactive microenvironment of specific cells and tissues (Balint et al., 2013), the development of scaffolds that combine piezoelectric properties and topographical cues known to induce specific cellular responses are of particular interest.

Poly(vinylidene fluoride) (PVDF) (-CH_2_-CF_2_-) is a thermoplastic polymer which is widely used in biomedical applications (Ahmed et al., 2014; Fernandes et al., 2019). This synthetic material shows a number of properties that makes it an ideal piezoelectric candidate for TE applications, including high biocompatibility and good mechanical performance (Motamedi et al., 2017a; Haddadi et al., 2018; Mokhtari et al., 2021). In addition, the piezoelectric response of PVDF and copolymers is the highest among polymer materials (Motamedi et al., 2017b; Li et al., 2019; Mokhtari et al., 2021).

A wide variety of structured scaffolds based on PVDF have been developed so far. One of the most widely used methodologies for the manufacturing of PVDF-based films on rigid or flexible large-area surfaces, is doctor blade (Ribeiro et al., 2018), which renders flat films of homogenous thickness. This PVDF architecture is the most used in sensor and actuator applications (Seminara et al., 2011). On the other hand, porous PVDF structures, such as porous membranes, can be produced by salt-leaching or phase separation methods (Ribeiro et al., 2018), among others. These structures have been proposed as candidates for the repair of a number of tissues, including as vessels, bones, and muscles (Ribeiro et al., 2018). Finally, PVDF scaffolds using electrospinning technology are composed of nano- or microfibers with controlled orientation (Maciel et al., 2017). The effect of such ECM-like topographies has shown to have a great impact on cell behavior, and this structuring approach has been consequently widely proposed for biomedical TE applications (Li et al., 2019; Mokhtari et al., 2021).

The physicochemical and biological characteristics of PVDF scaffolds strongly depend on their architecture and preparation methodology. In addition, studies have identified that these factors are important for the development of structured scaffolds for cardiovascular system repair (Durán-Rey et al., 2021).

Endothelial cells are important constituents of the blood vessels playing a critical role in the cardiovascular homeostasis (Sun et al., 2020). Furthermore, when the biomaterial is implanted, the interaction between the material and the endothelium, that is the cellular membrane formed by endothelial cells that line the inside of the heart and blood vessels, is a key factor that determines the outcome (Hauser et al., 2017). In most of the studies, this interaction has been investigated with human umbilical vein endothelial cells (HUVECs), that represent a widely used source of the endothelial cells for *in vitro* studies (Kocherova et al., 2019). Previous studies have demonstrated the importance of surface electric charges and of the piezoelectric activity of biomaterials on the adhesion of endothelial cells and on their function (Bouaziz et al., 1997; Hitscherich et al., 2016).

In this context, PVDF-based structures have been developed by doctor blade, salt-leaching, and electrospinning methodologies to generate films, porous membranes, and electrospun scaffolds with random and oriented architectures, in order to tune their physicochemical properties and biocompatibility with HUVECs.

## 2 Materials and methods

### 2.1 Materials

Poly(vinylidene fluoride), PVDF, 10/10 powder was obtained from *Solvay* (Brussels, Belgium). N,N-dimethylformamide (DMF) and absolute ethanol were obtained from *Merck* and Sodium Chloride (NaCl) was obtained from *Sigma*. All reagents and solvents were used as received.

### 2.2 Sample preparation

Several processing techniques and post-treatment protocols were employed to produce the different PVDF based structures.

#### 2.2.1 PVDF solution preparation

First, a 10 wt% solution of PVDF powder was dissolved in DMF under magnetic stirring. A slight heating at 30°C was applied during the first 30 min of dissolution to speed up the process and the solution was then allowed to cool under magnetic stirring until a homogeneous and transparent solution was obtained, which took no more than 3 h. The polymer concentration was defined in order to obtain a solution compatible with the processing techniques and conditions in order to obtain the membranes (Ribeiro et al., 2018).

#### 2.2.2 Films preparation

The PVDF solution was homogeneously distributed by doctor blade method (∼450 µm spacer) over clean glass substrates. The samples were then placed in an oven (JP Selecta, Model 2000208) for 10 min at a temperature of 210°C for polymer melting and complete removal of the solvent. Next, films were removed from the oven and allowed to cool at room temperature. Samples with an average thickness of ∼24.53 µm were obtained.

#### 2.2.3 Porous membrane preparation

Porous membranes were prepared by a salt-leaching method. NaCl particles with an approximate size of 2 µm were added to the PVDF solution in a proportion of 10:3 (w:w) PVDF:salt. After a 1 h of energetic stirring, a homogeneous mixture was obtained. The resulting solution containing the dispersed NaCl was poured over glass Petri dishes and the solvent was evaporated at room temperature. The PVDF/NaCl membranes were then washed by distilled water bath at room temperature until the washing solution showed constant electrical conductivity values indicating the completely removal of the NaCl. Finally, the obtained porous membranes were placed in an airing chamber and dried for 24 h. Samples with an average thickness of ∼252.67 µm were obtained.

#### 2.2.4 Electrospun fiber mats

The PVDF solution was transferred to 10 ml disposable syringes fitted with a blunt steel needle with an inner diameter of 500 µm and placed in a syringe pump (*New Era NE-1000*). Electrospinning was conducted using a high voltage power supply (*Glassman PS/FC30P04*) set at 15 kV and the syringe was pumped with a flow rate of 0.5 ml h^−1^. The resulting randomly oriented electrospun PVDF 10/10 membranes were collected on a grounded 20 × 15 cm static plate collector placed 15 cm from the needle. The processed membranes are identified in the following as ES-NO membranes.

Oriented PVDF membranes were obtained after a process similar to the one described above, except for the use of a grounded rotating drum collector, which was set at a speed of 1,500 rpm. The processed membranes are identified as ES-O membranes. Samples with an average thickness of ∼137.5 µm and ∼26.53 µm were obtained for the randomly oriented and oriented samples, respectively.

### 2.3 Sample characterization

#### 2.3.1 Physicochemical characterization

Scanning electron microscope (SEM) JEOL JSM-7000F was used to obtain the surface and cross section morphologies of the PVDF membranes. From these images, the pore size and fiber diameter distributions were determined using the software ImageJ (Brito-Pereira et al., 2018).

Pycnometer was used to measure the porosity of the samples by liquid displacement. The pycnometer was filled with ethanol, the weight was measured and labelled as W_1_. The sample, which weight was (W_S_), was immersed in ethanol. Once the sample was saturated, additional ethanol was added to completely fill the volume of the pycnometer, and the system was weighed (W_2_). Finally, the sample was taken out of the pycnometer. W_3_ was the weight of the system with ethanol. The porosity of the sample was obtained as the average of three values according to Eq. 1 (Brito-Pereira et al., 2022):

$$\varepsilon =\frac{W_{2\;}-W_{3\;}-W_{S}}{W_{1\;}-W_{3}}$$ (1)

Absolute ethanol is a non-solvent for PVDF, so it was used as displacement liquid since it can penetrate the pores without inducing swelling, shrinking or sample degradation.

The wettability of the samples was evaluated with an OCA20 instrument by the static sessile drop method with ultrapure water. For that, a water drop was placed onto the surface of the samples and the SCA20 software was used to measure the contact angle. The mean contact angle and standard deviation were calculated from the measurement at six different positions for each sample.

The different PVDF structures were subjected to stress-stress measurements using a *Shimadzu AD-IS* universal testing set up with a load cell of 50 N. The dimensions of the samples were 15 mm long and 10 mm wide. Average sample thickness for the samples, as measured using a Fischer Dualscope MPOR were ∼24.53 µm (films), ∼252.67 µm (porous membranes), ∼137.5 µm (electrospun randomly oriented scaffolds), and ∼26.53 µm (electrospun oriented scaffolds). Samples were stretched at a rate of 5 mm/min^−1^ for films and 1 mm/min^−1^ for the remaining scaffolds. Three samples of each structure of PVDF were used to perform the measurements, and the ES-O PVDF membranes were stretched along and in 90° regarding the direction of the fibers [ES-O (90°)].

Infrared measurements (FTIR) and differential scanning calorimetry (DSC) were also performed to evaluate polymer phase and thermal behaviour and degree of crystallinity, respectively. FTIR was performed using a Jasco FT/IR 4100 (Agilent 4300) system in attenuated total reflectance mode (ATR) from 4000 to 650 cm^−1^. A resolution of 4 cm^−1^ was used to obtain FTIR spectra after 64 scans. The 763 and 840 cm^−1^ absorption bands are attributed to α and β phases of PVDF, respectively (Martins et al., 2014). The β phase content of the produced samples was calculated according Eq. 2.

$$F\left(\beta \right)=\frac{A_{\beta }}{\left({K_{\beta /}K}_{\alpha }\right)A_{\alpha }+A_{\beta }}\times 100$$ (2)

where, F(β) is the β phase content; A_α_ and A_β_ the absorbance at 763 and 840 cm^−1^ respectively; $K_{\alpha }$ and $K_{\beta }$ the adsorption coefficients at the respective wavenumber, with values of 6.1 × 10^4^ and 7.7 × 10^4^ cm^2^ mol^−1^, respectively (Martins et al., 2014).

Differential scanning calorimetry (DSC) studies were performed in a Mettler Toledo DSC822e apparatus using a heating rate of 10°C.min^−1^. The samples were cut into small pieces and placed into 40 µL aluminum pans. Through the obtained thermograms, the degree of crystallinity (χc) was determined by Eq. 3 (Maciel et al., 2017):

$$X_{c}\left(\%\right)=\frac{{∆H}_{f}}{{x∆H}_{\alpha }+{y∆H}_{\beta }}\times 100$$ (3)

where, according to FTIR-ATR measurements, x and y are the fraction of α and β phases of PVDF, respectively. ∆H_f_ is the melting enthalpy of the samples and ∆H_α_ (93.04 J g^−1^) and ∆H_β_ (103.4 J g^−1^) are the melting enthalpies of the α and β phases of a 100% crystalline sample of PVDF (Benz and Euler, 2003).

#### 2.3.2 Cytotoxicity assay

The ISO 10993-5 standard test was used to evaluate the indirect cytotoxicity of the different samples. For this purpose, L929 adipose cells were cultured in 75 cm^2^ cell culture flask in a humidified environment at 37°C with 5% CO_2_, using Dulbecco’s modified Eagle’s medium (DMEM, Biochrom), with 4.5 g.L^−1^ glucose, 10% fetal bovine serum (FBS, Biochrom) and 1% (v/v) penicillin/streptomycin solution (P/S, Biochrom).

Before the assay, the samples were cut in 1.5 cm^2^ and sterilized using ultraviolet radiation for 1 h on each side. After that, samples were washed with sterile phosphate-buffered saline solution (PBS, pH 7.4).

After sterilization, samples were incubated in DMEM in a 24-well tissue culture plate for 24 h. Simultaneously, 5 × 10^4^ cell/mL cells were seeded in a 96-well plate and allowed to adhere for 24 h. After that, the culture medium in the 96-well culture plate was removed and replaced with 100 µL of the culture medium, which was in contact with the PVDF samples. As positive control dimethylsulfoxide (DMSO) at 20% and a negative control (fresh DMEM) were used. After 72 h (3-(4,5-dimethylthiazol-2-yl)-5-(3-carboxymethoxyphenyl)-2-(4-sulfophenyl)-2H-tetrazolium) (MTS, Promega) was used to quantify cell survival. Briefly, the culture medium was replaced with fresh medium containing MTS solution in a 1:5 ratio and cells were incubated for 2 h. After that, a spectrophotometric plate reader (Biotech Synergy HT) at 490 nm was used to measure the optical density. The cell viability was calculated according to Eq. 4 (Fernandes et al., 2019):

$$cell\;viability\;\left(\%\right)=\frac{absorbance\;of\;sample}{negative\;control\;absorbance}x100$$ (4)

### 2.4 Cell culture assays

For the *in vitro* assays, PVDF samples (films, porous membrane, ES-NO and ES-O) were cut into circular shapes with 13 mm of diameter. For sterilization, the samples were washed 5 times in PBS 1x solution for 5 min each. Subsequently, the samples were exposed to ultraviolet (UV) light for 2 h (1 h each side). Finally, 24-well cell culture plates were used to place the samples.

Human Umbilical Vein Endothelial Cells (HUVEC’s cells, ATCC) were grown in 75 cm^2^ cell-culture flask and cultured with endothelial cell growth medium (*Pan Biotech*). A humidified air containing 5% CO_2_ atmosphere was used to incubate the flask at 37°C. The culture medium was changed every 2 days. Accutase (*Grisp*) was used to detach the cells when they reached 80%–90% confluence.

For proliferation tests, HUVEC’s cells were seeded on the different samples at a density of 7,500 cells. cm^−2^. The drop method was used to allow the cell attachment on the different samples. Samples were incubated at 37°C in a saturated humidity atmosphere containing 95% air and 5% CO_2_. After selected times, cell viability and immunofluorescence microscopy were used to analyse the samples.

For the proliferation assays, the MTS assay was used to evaluate the cell viability of HUVEC’s on the different materials. This method can be used as indirect assay to evaluate cell proliferation, according to the increase of cell viability. The MTS assay was carried out after 1 and 4 days. At these time points, the cell/films were transferred to new wells and fresh medium containing MTS solution (1:5 proportion of medium) was added. The plate was incubated at 37°C for 3 h. Finally, a microplate reader (*Biotech Synergy HT*) was used to measure the absorbance at 490 nm. Experimental data were obtained from four replicates of each sample. Results were expressed as mean ± standard deviation. All quantitative data were analyzed using GraphPad by Dotmatics. The results were analyzed statistically using the *t*-test. Differences were considered statistically significant when *p*-value < 0.01.

After 4 days, fluorescent labelling was used to staining the actin and nucleus of the cells in the different samples. For that, the medium from each well was removed, the samples were washed with PBS 1x and fixed with 4% formaldehyde for 10 min at 37°C in a 5% CO_2_ incubator. After fixation, PBS 1x (three times) was used to wash the samples, and they were permeabilized with 0.1% Triton X-100 for 10 min at room temperature. After that, all samples were incubated for 45 min at room temperature in 0.1 μg ml^−1^ of Phalloidin–Tetramethylrhodamine B isothiocyanate (TRITC, *Sigma-Aldrich*). Then, 1 μg ml^−1^ of DAPI (*Sigma-Aldrich*) was used to incubate the samples for 5 min. Afterwards, the samples were washed again with distilled water (two times). Finally, fluorescence microscopy (Olympus BX51 Microscope) was used to visualise the samples.

## 3 Results and discussion

### 3.1 Physicochemical characterization

**FIGURE 1:**
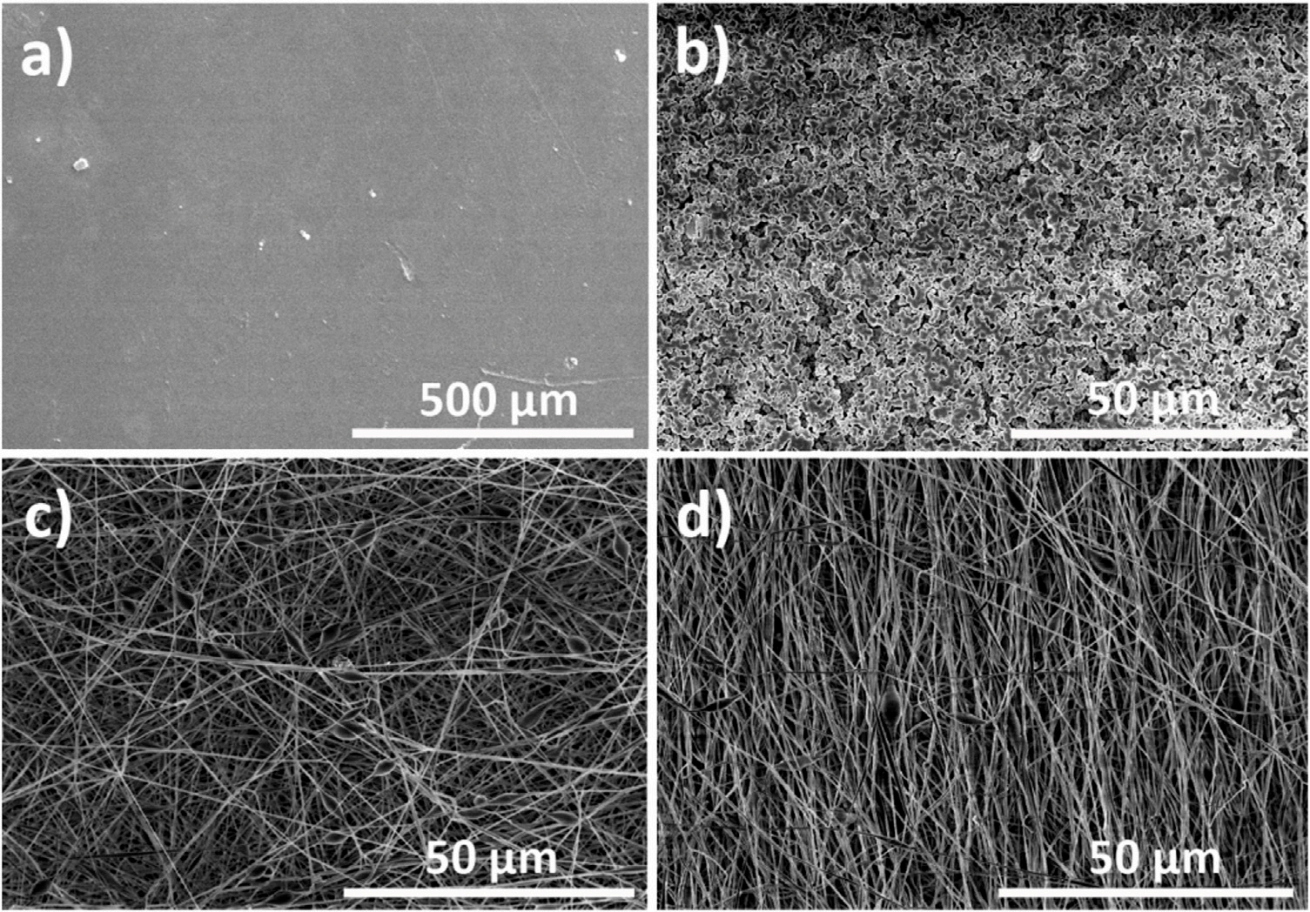
Representative SEM images of the processed PVDF structures, namely: **(A)** films, **(B)** porous scaffold, **(C)** electrospun randomly-oriented fibers, and **(D)** electrospun oriented fibers.

In order to verify the morphology of the PVDF membranes after processing, representative SEM images of the PVDF membranes were obtained (Figure 1). From these images, the main pore size and fiber diameter distributions were determined using the image analysis software ImageJ.

The images reveal the different characteristics of each PVDF-based structure. First, PVDF films produced by doctor blade lack porosity, while PVDF membranes prepared by salt-leaching method are characterized by pores of approximately 2 µm. It is to notice that the used methodology allows the control of the pore size by tuning the size of the NaCl particles added to the mixture. However, structures manufactured with salt-leaching do not present an interconnected network of pores, and frequently show irregular and non-reproducible architectures (Cardoso et al., 2015; Ribeiro et al., 2018). Finally, fibrous PVDF scaffolds with randomly distributed (ES-NO) and oriented fibers (ES-O) produced by electrospinning contained average fiber widths of 634.7 ± 31 and 598.1 ± 27 nm, respectively. These results are consistent with previously published data of PVDF electrospun scaffolds (Brito-Pereira et al., 2021). The fact that ES-O scaffolds had a smaller average fiber diameter than ES-NO scaffolds can be attributed to the stretching of the oriented fibers during their collection in the rotating drum (Ribeiro et al., 2010).

**FIGURE 2:**
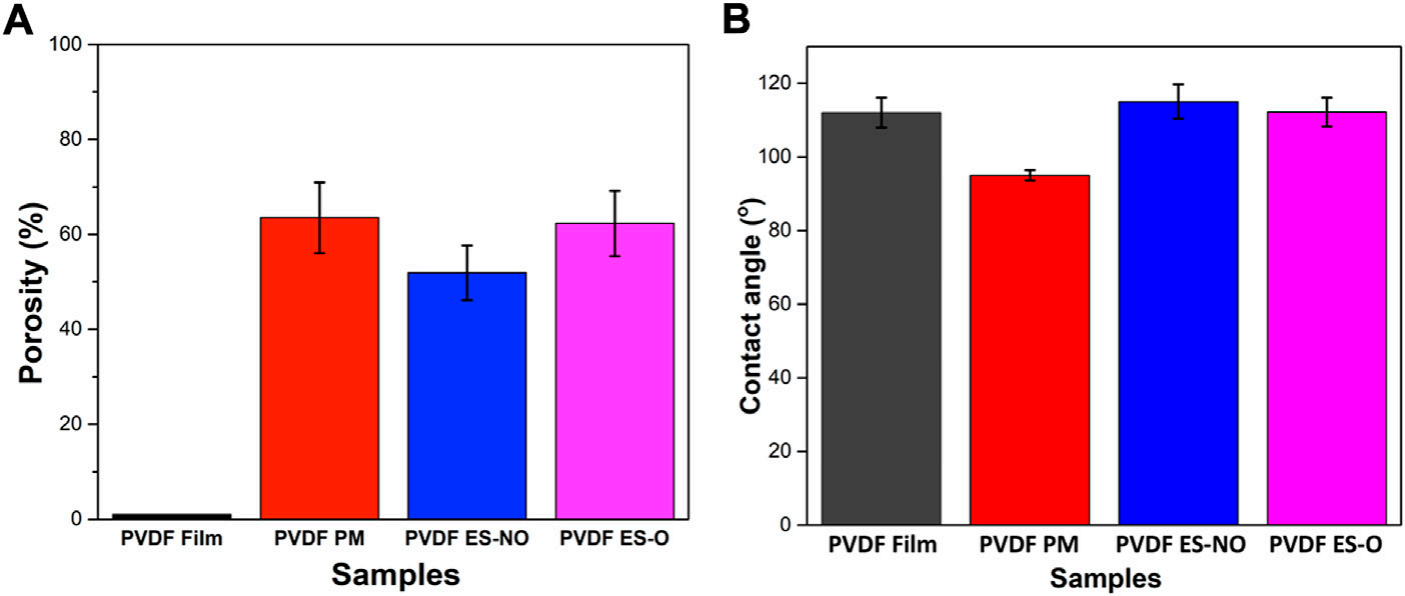
**(A)** Porosimetry results of the PVDF film, porous membrane (PM), electrospun randomly-oriented membranes (ES-NO) and electrospun oriented membranes (ES-O). **(B)** Representative contact angle of the PVDF film, PM, ES-NO and ES-O.

Independently of the processing method and porous membrane architecture, the mean degree of porosity was above 50% (Figure 2A). Porosity values of 63.51% ± 7.45%, 51.9% ± 5.8%, and 62.3% ± 6.89% were calculated for porous membranes, ES-NO, and ES-O scaffolds, respectively. Nevertheless, the degree of porosity, as well as pore structure and fiber widths, can be further tune according to the selected processing parameters and methodology (Bhardwaj and Kundu, 2010; Ribeiro et al., 2010; Ribeiro et al., 2018).

Wettability is a key parameter affecting cell adhesion, proliferation and differentiation (Razafiarison et al., 2018; Kitsara et al., 2019). The water contact angle of the processed films, porous scaffolds, ES-NO and ES-O membranes were 111.98 ± 4.12°, 94.99 ± 1.43°, 115.01 ± 4.68°, and 112.19 ± 3.90°, respectively (Figure 2B). Note that hydrophobic surfaces show water contact angles higher than 90°, while values below 90° correspond to hydrophilic surfaces. In turn, materials with water contact angle values above 150° and below 10° are termed superhydrophobic and superhydrophilic, respectively (Otitoju et al., 2017). Therefore, all PVDF membranes showed a hydrophobic behavior, though slight differences in the surface wettability are observed depending on the topography of the membranes. Electrospun membranes show a more hydrophobic behavior than other membrane structures (Ahmed et al., 2014; Szewczyk et al., 2018). In particular, PVDF porous membranes show a relatively large pore size, which causes the liquid to percolate into the pores and, therefore, slightly lowers the contact angle value (Szewczyk et al., 2018).

**FIGURE 3:**
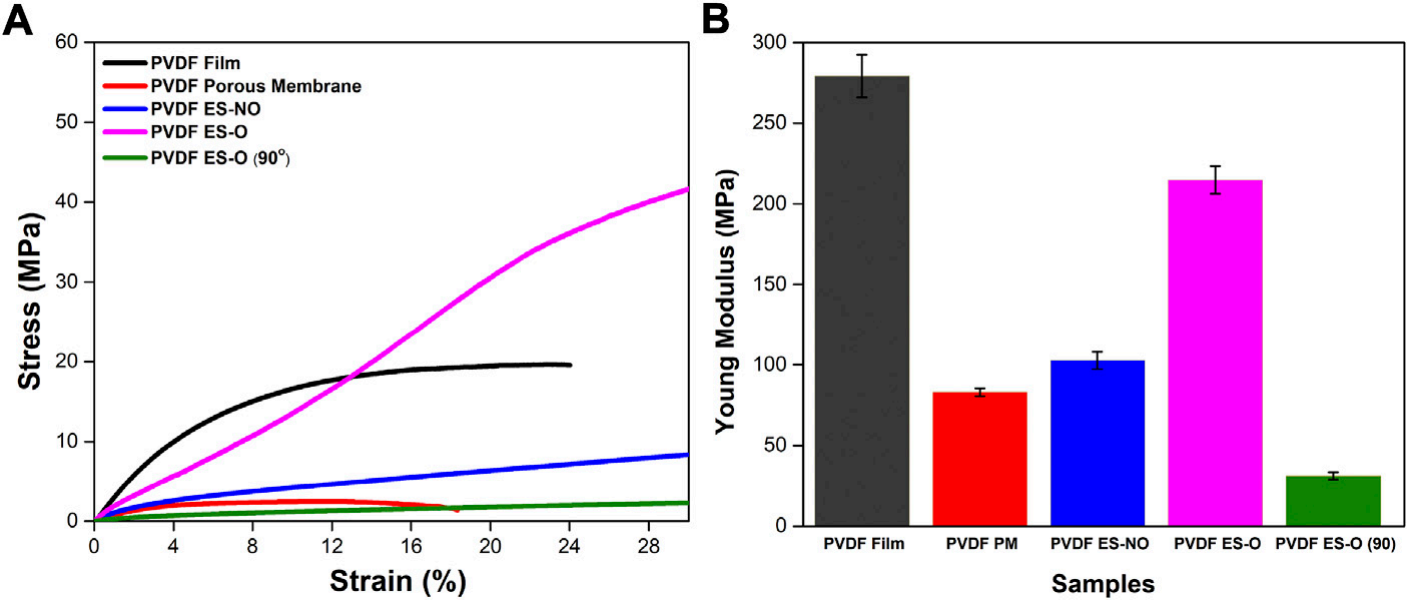
**(A)** Stress-strain curves up to 28% of strain for the PVDF film, porous membrane (PM), electrospun randomly-oriented membranes (ES-NO), electrospun oriented membranes (ES-O) and 90° electrospun oriented membranes [ES-O (90°)]. **(B)** Young’s modulus with mean and standard deviation.

The mechanical properties of biomaterials for TE applications are particularly relevant for the development of safe and durable solutions. Although the PVDF structures are composed of the same material, their mechanical stability varies depending on the geometry and manufacturing methodology. Therefore, stress-strain measurements were performed in all PVDF samples. PVDF is a thermoplastic elastomer which characteristic stress-strain mechanical response (Figure 3) feature a first linear elastic regime, where the structure returns to its original shape when stress ceases, followed by a plastic regime after yielding, where the material does not recover its original shape, undergoing permanent deformation. Finally, the rupture stress-strain is reached if the sample is stretched further. On the other hand, materials can be divided mainly into ductile, which have a typical elastic and plastic zone that is perfectly differentiated, and brittle materials, which typically present little or no plastic deformation. Hooke’s law is applied to calculate the Young’s modulus from the linear regime of the curves (Brito-Pereira et al., 2021).

All analyzed PVDF structures feature stress-strain curves characteristic of ductile materials (Figure 3A). However, there are significant differences in the Young’s moduli and in the mechanical properties of the materials depending on the architecture of the structures (Figure 3B).

PVDF films are stiff with a flat and dense morphology and characterized by a Young’s modulus higher than the PVDF porous membranes (Cardoso et al., 2015), being 279.22 ± 13.27 and 83.01 ± 2.52 MPa, respectively. On the other hand, mechanical analysis of films and porous membranes showed a maximum elongation of approximately 24% and 18%, respectively, which correspond to their rupture stress-strain. Films have generally higher stress-strain values than porous membranes, as the mechanical response of the latter is first determined by the deformation of the porous structure. Furthermore, stress-strain values of porous membranes have a higher variability due to the random distribution of the pores (Cardoso et al., 2015).

Regarding the electrospun membranes, samples deformed along the main fiber direction (ES-O (0°)) showed a higher value of the effective Young’s modulus (214.77 ± 8.64 MPa), followed by ES-NO (102.73 ± 5.39 MPa) and ES-O (90°) (31.16 ± 2.26 MPa), respectively. These results are consistent with previous studies, where the Young’s modulus decreases with increasing angle between fiber orientation and strain direction (Heo et al., 2011; Wang et al., 2014; Maciel et al., 2017). This is due to fiber reorientation and collapsing of pores as fibers are recruited toward the direction of applied strain (Heo et al., 2011). On the contrary, electrospun membranes showed an elongation larger than 28%. Previous studies indicated that the elongation at break of electrospun PVDF membranes could reach 142%, displaying good ductility (Cai et al., 2019). In fact, ES-O (0°) showed a yield strain and stress larger than ES-NO and ES-O (90°). This is because the stress is applied mostly along the fiber direction in the case of ES-O (0°), while in ES-NO and ES-O (90°) the stress is first devoted to the reorientation of the fibers along the stretching direction (Maciel et al., 2017). In this way, the mechanical properties of the material are dependent on the orientation of the fibers and, therefore, can be adjusted to the specific biomedical application.

**FIGURE 4:**
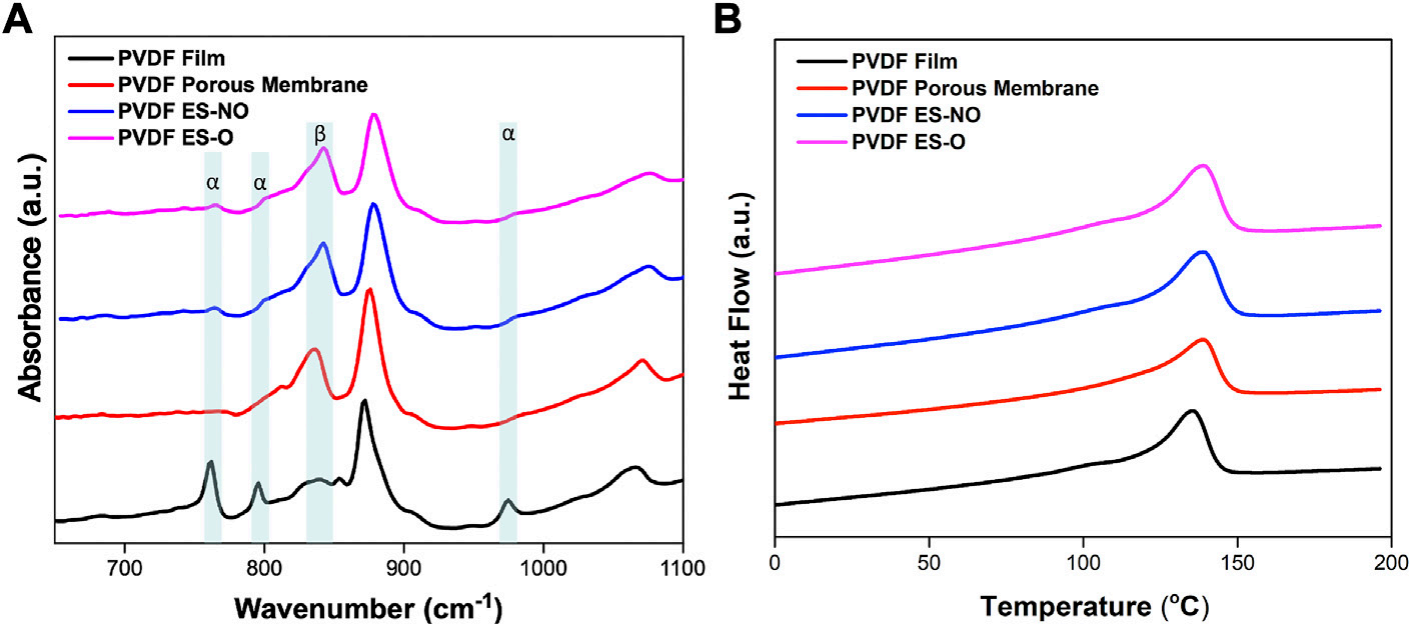
**(A)** Representative FTIR spectra of PVDF film, porous scaffold, electrospun randomly-oriented membranes (ES-NO) and electrospun oriented membranes (ES-O). **(B)** Representative DSC thermograms of the PVDF film, porous membrane, ES-NO and ES-O.

Further, FTIR analysis show that the processing method used to generate PVDF structures has a great influence on the crystalline phase of the scaffolds (Figure 4A). PVDF films processed at high temperatures display higher content of *a*-phase (762, 796 and 975 cm^−1^), as the processing temperature is one of the key factors determining the crystalline structure of the polymer (Ribeiro et al., 2010). On the other hand, when processed at room temperature, structured PVDF (porous, ES-NO and ES-O) are characterized by having a higher content of electroactive *β*-phase (840 cm^−1^). Table 1 summarizes the electroactive *β*-phase content of the different samples, calculated after Eq. 2, showing that the samples processed at room temperatures show an electroactive *β*-phase content above 88%, whereas for the films obtained after high temperature processing it is reduced below 7%.

**TABLE 1:** β-Phase content, melting temperature and degree of crystallinity of the produced PVDF samples, as obtained from FTIR and DSC respectively.

	β phase content	T_m_ (°C)	X_c_ (%)
PVDF Film	6.3 ± 0.1	135.7 ± 3.5	49.1 ± 2.1
PVDF PM	99.0 ± 0.8	138.8 ± 4.0	38.7 ± 1.4
PVDF ES-NO	90.0 ± 1.8	138.7 ± 4.1	51.3 ± 3.6
PVDF ES-O	88.9 ± 1.6	138.9 ± 3.7	50.4 ± 2.5

DSC measurements were performed in all membranes (Figure 4B) and just slight variation are observed in the melting transition (*T*_*M*_), which occurs at 135.7 ± 3.5°C for PVDF films and 138.8 ± 4.0°C for the remaining membranes. The different is fully attributed to the crystallization conditions: whereas the films are processes from the melt, the membranes are the final result of a solvent evaporation process. The melting enthalpy obtained from the area underneath the melting peak of each thermogram, and the degree of crystallinity were calculated after Eq. 3, resulting in 49.1% ± 2.1% for films, 38.7% ± 1.4% for porous membranes, 51.3% ± 3.6% for randomly oriented fibers and 50.4% ± 2.5% for oriented fibers (Table 1).

It can be concluded that the degree of crystallinity slight differs for all different membranes with a mean value between 40% and 55%, which is typical for PVDF membranes and that the decrease of the β phase content of the films does not induces significant changes the degree of crystallinity (Ribeiro et al., 2010). Thus, the variations of the mechanical properties are just attributed to the different morphological features of the PVDF membranes and not in differences in the degree of crystallinity, which agrees with the literature (Brito-Pereira et al., 2021).

### 3.2 Cytotoxicity assay

**FIGURE 5:**
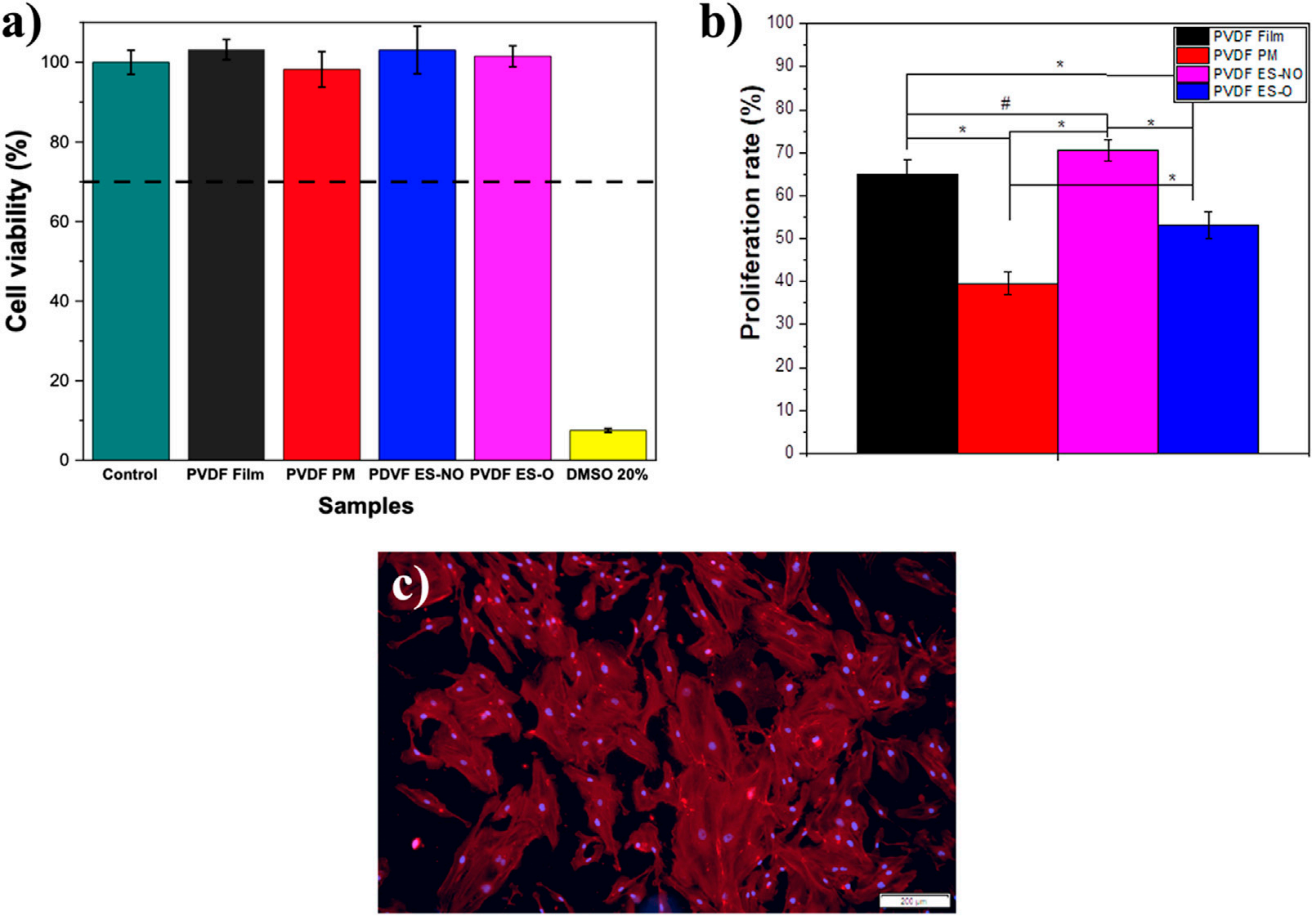
**(A)** Cell viability of control L929 cells, in contact with the extraction media of PVDF films, porous membrane (PM), electrospun randomly-oriented membranes (ES-NO), and electrospun oriented membranes (ES-O) (relative metabolic activity was presented as the percentage of the negative control with *n* = 4 ± standard deviation). **(B)** Proliferation rate determined using the MTS assay on HUVEC’s cells seeded on the different PVDF samples after 4 days. The proliferation rate was calculated regarding the cells growing on the material after 24 h of cell adhesion. Results are expressed as mean ± standard deviation, *n* = 4. #*p* < 0.002 and *p < 0.0001. **(C)** HUVEC’s cells after 4 days of cell culture on PVDF films.

Low cytotoxicity is essential for biomaterials for TE applications. PVDF has been used as a scaffold for different TE applications (Motamedi et al., 2017a; Ribeiro et al., 2018b; Fernandes et al., 2019). Nevertheless, since different processing techniques were used and in order to prove that they did not affect the toxicity of the produced samples, an indirect cytotoxicity assay was carried out. For this reason, analysis of indirect toxicity using L929 adipose cells was used to assess the cytotoxicity of all generated structures (Figure 5A). According to the ISO 10993-5, when the cell viability suffers a reduction larger than 30%, the samples are considered cytotoxic. All PVDF morphologies showed cell viability values close to 100%, independently of their architecture and processing conditions, which is in concordance with previous studies (Ribeiro et al., 2018c).

### 3.3 Cell proliferation

HUVEC cell viability after incubation with extraction media, and proliferation after seeding on the different PVDF samples were evaluated through MTS assay (Figure 5B).

None of the generated PVDF scaffolds did show toxicity in indirect culture conditions (Figure 5A). In addition, when seeded on the structured materials HUVEC cells did proliferate, being the highest proliferation ratios observed in cells seeded on PVDF films and ES-NO membranes.

Fiber diameter and porosity affect cell adhesion, ingrowth and proliferation, and for that reason, these are parameters must be carefully considered for the production of materials for tissue engineering applications. Previous studies have demonstrated an increase in the initial attachment of endothelial cells on fibers with lower fiber diameters (Bergmeister et al., 2013). In our experiments, scaffolds of PVDF randomly and oriented fibers display similar fiber diameter and porosity, so the differences observed on HUVECs proliferation are most likely caused by fiber orientation. Nevertheless, the specific response to fibrous topographies has been shown to be highly affected by the composition of the fibers and environmental factors (Veleva et al., 2009; Cui et al., 2017; Yu et al., 2020).

In order to evaluate the cytoskeleton morphology of the cells and to verify cell viability, immunofluorescence tests were also performed in the films (Figure 5C). It is shown that all PVDF samples promote the formation of HUVEC monolayers composed of well-spread cells.

Overall, it is demonstrated that the electroactive PVDF based scaffolds with different morphologies can be used for HUVECs cell culture, allowing the further design of vascular implants.

## 4 Conclusion

PVDF is a thermoplastic electroactive polymer which is widely used in TE applications. It has been shown that, when structured, PVDF displays different characteristics depending on the architecture of the scaffold and processing methodology. Thus, PVDF has been processed in the form films, porous and electrospun membranes. It is shown that all PVDF membranes feature stress-strain curves typical of a ductile material. However, films and porous membranes present a maximum elongation of approximately 24% and 18%, respectively, while electrospun membranes show an elongation greater than 28%. The crystalline phases of the PVDF samples is affected by the processing method, where PVDF films mainly crystallize in the non-polar *a*-phase, while the remaining PVDF membranes are characterized by having high electroactive *β*-phase contents. The processing temperature is one of the key factors that affect the crystalline structure of PVDF, as PVDF film that was processed at high temperatures, and the remaining PVDF membranes (porous and electrospun membranes) were processed at room temperature. Despite the methodology employed for the design of PVDF membranes, no significant variation was observed in the melting transition, and all PVDF showed hydrophobic behavior. However, surface wettability is affected by the morphology of the PVDF membrane, having a higher hydrophobic behavior the electrospun membranes due to its surface roughness. Finally, cytotoxicity assay proved that, independently of the topography, PVDF maintained high levels of cell viability, and promoted HUVEC cell proliferation, being this higher on the film and electrospun randomly-oriented membranes. All these material characteristics should be taken into account when designing a PVDF membrane for TE applications, including but not limited to cardiovascular system. The results demonstrate that PVDF is a promising candidate to be used for cardiovascular applications.

## Data Availability

The original contributions presented in the study are included in the article/Supplementary Material, further inquiries can be directed to the corresponding author.
